# Self-Organization Provides Cell Fate Commitment in MSC Sheet Condensed Areas via ROCK-Dependent Mechanism

**DOI:** 10.3390/biomedicines9091192

**Published:** 2021-09-10

**Authors:** Peter Nimiritsky, Ekaterina Novoseletskaya, Roman Eremichev, Natalia Alexandrushkina, Maxim Karagyaur, Oleg Vetrovoy, Nataliya Basalova, Anastasia Khrustaleva, Alexander Tyakht, Anastasia Efimenko, Vsevolod Tkachuk, Pavel Makarevich

**Affiliations:** 1 Institute for Regenerative Medicine, Medical Research and Education Center, Lomonosov Moscow State University, 119192 Moscow, Russia; novoseletskaya.es@gmail.com (E.N.); romaneremichev@gmail.com (R.E.); n.alexandrushkina@gmail.com (N.A.); darth_max@mail.ru (M.K.); basalova.natalia@gmail.com (N.B.); AEfimenko@mc.msu.ru (A.E.); tkachuk@fbm.msu.ru (V.T.); pmakarevich@mc.msu.ru (P.M.); 2 Faculty of Medicine, Lomonosov Moscow State University, 119192 Moscow, Russia; 3 Laboratory of Regulation of Brain Neuron Functions, Pavlov Institute of Physiology, Russian Academy of Sciences, 199034 Saint Petersburg, Russia; vetrovoyov@infran.ru; 4 Department of Biochemistry, Faculty of Biology, Saint-Petersburg State University, 199034 Saint Petersburg, Russia; 5 Department of the Bioinformatics, Institute of Gene Biology, Russian Academy of Sciences, 119334 Moscow, Russia; nastia.khrust@gmail.com; 6 Center for Precision Genome Editing and Genetic Technologies for Biomedicine, Institute of Gene Biology, Russian Academy of Sciences, 119334 Moscow, Russia; a.tyakht@gmail.com

**Keywords:** self-organization, self-patterning, mesenchymal condensation, cell sheets

## Abstract

Multipotent mesenchymal stem/stromal cells (MSC) are one of the crucial regulators of regeneration and tissue repair and possess an intrinsic program from self-organization mediated by condensation, migration and self-patterning. The ability to self-organize has been successfully exploited in tissue engineering approaches using cell sheets (CS) and their modifications. In this study, we used CS as a model of human MSC spontaneous self-organization to demonstrate its structural, transcriptomic impact and multipotent stromal cell commitment. We used CS formation to visualize MSC self-organization and evaluated the role of the Rho-GTPase pathway in spontaneous condensation, resulting in a significant anisotropy of the cell density within the construct. Differentiation assays were carried out using conventional protocols, and microdissection and RNA-sequencing were applied to establish putative targets behind the observed phenomena. The differentiation of MSC to bone and cartilage, but not to adipocytes in CS, occurred more effectively than in the monolayer. RNA-sequencing indicated transcriptional shifts involving the activation of the Rho-GTPase pathway and repression of SREBP, which was concordant with the lack of adipogenesis in CS. Eventually, we used an inhibitory analysis to validate our findings and suggested a model where the self-organization of MSC defined their commitment and cell fate via ROCK1/2 and SREBP as major effectors under the putative switching control of AMP kinase.

## 1. Introduction

Multicellular species consist of a vast variety of cells organized into specialized tissues that form the unique architecture of each organ. The complexity of multicellular formations unfolds within the framework of a developmental program that provides the reproducibility and robust control of morphogenetic events. The primary structural information confined within the protein-coding part of the genome has long been considered the main instructive force behind the development from primordial structures to mature tissues and body parts. Indeed, aberrations of the genomic structure are associated with nonviable or severely distorted phenotypes.

The structure and functions of a mature tissue are defined by the sum of specific expression profiles and intrinsic programs of its cells. However, extrapolating individual cell’s properties to a higher organizational level of tissue, we encounter a complex emergent system. The emergence feature is a behavior of a system collectively defined by entities that do not demonstrate this behavior in a separated state. To simplify this, we may say: “what parts of a system do together they would never do alone?” [1].

This concept has been successfully applied to the process of determination during embryonic development when big choices are made. “Head or tail”, “left or right side”, “budding or dispersing” and “limb or stub” decisions are made and supported by distinct instructive forces [2]. These instructions emerge from intercellular interactions that include differential adhesion, polarity, positional signals, etc. The subsequent cell specialization and morphogenesis occur under a stringent control provided by intrinsic properties and extrinsic instructions for each cell [3].

To an independent observer, this is best described as tissue “self-organization” de novo from a mass of cells where decisions (once uninterrupted) are made in an optimal manner to provide rapid and finely tuned unfolding of the mature structure [4]. Cells of an appropriate potency may self-organize ex vivo to very complex organotypic entities known as organoids [5,6]. Embryonic stem cells (ESC) can give rise to optical cup, multilayered cortex, crypt-like intestinal structures and many others [7,8]. This suggests that, once a critical mass of cells and interactions are present and the necessary genes expression is on, ESC can form organ-like structures relying on their autonomous properties. The above-mentioned critical mass of cells highlights the importance of diversity and magnitude of interactions between them to emerge as a self-organizing complex system, e.g., in *Hydra magnipapillata*, known for their amazing regenerative ability, just 250–300 epithelial cells are required to form a new individual from a shattered one [9]. This is the minimal number of cells sufficient for the self-organization of a primitive spherical shell, and a piece of tissue too small to form it will fail to give rise to a new hydra.

In developmental biology, self-organization is conditionally divided into three basic processes [10]:

(1) Self-assembly is the spatiotemporal positional redistribution of cells due to local rules of interaction, such as differential adhesion and motility. This results in the spontaneous formation of a new structure by differential aggregation or the rearrangement of cells with their preferential positions defined by interactions sufficient to generate a local instruction for a specific cell type. In most cases, no drastic change of the cell program occurs, and this is sometimes termed the “self-sorting” of cells.

(2) Self-patterning is a more complex process when local environments influence the cell status (potency, phenotype and viability) independently of external cues. Self-patterning may occur in homogenous cell aggregates (spheroids and sheets), and vivid examples include skin patterns in fish, the spacing of feathers and hairs controlled autonomously by dynamic self-regulating rules. Overall, during this process, an initially homogeneous population of cells becomes heterogeneous due to spatial and temporal cues.

(3) Self-morphogenesis is a large-scale process involving changes of the form, tubulogenesis, modification of mechanical properties (viscosity and stiffness) and emergence of a specific 3D architecture. This is defined by the tension, traction, cell migration and stabilization of a structure to gain relative independence from external forces. Briefly, tissue that has emerged as a result of self-morphogenesis is capable of supporting its structure, relying only on newly formed internal signals, interactions and properties.

Thus, understanding how and why individual cells make a collective decision and unite in tissues is well-established in developmental biology. Despite the known arguable points and gaps of knowledge, we have a set of definitions that one may use to describe and dissect the observations made in this field.

After birth, the majority of morphogenetic events in the human body besides physiological growth and puberty is driven by tissue loss, trauma or disease—all of which can be termed “damage”. In response to injury, the human organism has a significant incline towards fibrosis, and the full restoration of tissue architecture is typically scarce and confined to a few organs (spleen, uterine endometrium and bone) or occurs after a minor injury (e.g., superficial skin wound) [11]. This is conventionally explained by the loss of cellular plasticity in most organs, a reduced potency of adult stem cells or their failure to properly respond to morphogenetic stimuli (growth factors or cytokines) released during damage and healing in abundance. However, we believe that self-organization and its mechanisms represent a fruitful field to establish approaches for successful tissue repair in the adult human body. The restoration of tissue architecture after damage requires an increasing complexity of cellular interactions for spatial self-organization and rearrangement to anisotropic microenvironments. The latter define and support the local rules of tissue formation partially resembling development in utero. In contrast to healthy tissues with complex architecture, scars in most organs present uniform, poorly organized structures. Therefore, a tissue- or species-specific tendency to complete regeneration or scarring may be associated with the specific features of intercellular interactions during self-organization after damage [12].

The cells of the embryonic mesenchyme are the major organizers and source of instructive input during histo- and organogenesis [13]. Their self-organization by condensation is crucial for the normal development of bone [14]; cartilage [15]; teeth [16]; tendons [17]; lungs [18,19]; muscles and the majority of parenchymatous organs (kidneys, liver, etc.) [20,21,22,23]. Condensed mesenchyme gains a morphological integrity, providing the synchronization of cells’ responses to external stimuli. Postnatal descendants of embryonic mesenchymal cells—mesenchymal multipotent stromal cells (MSC)—also possess an ability to self-organize. Adult fractured bone repair depends on MSC aggregation, which resembles mesenchymal condensation during development [24,25]. This is believed to be the crucial step in the commitment of adult MSC towards the osteogenic lineage required to provide a successful and unanimous response to bone morphogens [26]. Thus, our understanding of the MSC role in postnatal human body repair is related to their ability or failure to effectively self-organize for the restoration of tissue lost due to damage.

The concept of regeneration as a part of the development process ongoing throughout the lifetime has become more recognized [27]. Among developmental biologists, the phenomenon of self-organization is regarded as a mechanism underlying the formation of organ microarchitecture de novo [10,28]. Furthermore, self-organization is considered a possible basis for the developmental approach in contemporary tissue engineering and regenerative medicine (TERM) [29,30,31]. Therefore, dissecting the possible role of self-organization in post-traumatic regeneration creates a new prospect for future applications in therapy.

We have previously reported that spontaneous self-organization occurs in cell sheets (CS)—minimal tissue-engineered constructs from adult MSC [32]. This intrinsic program of MSC might be an attractive subject for the better understanding of the events taking place after damage and the prevention of scarring. Briefly, once human MSC are capable of self-organization, we may target the relevant genes and pathways known from embryonic development to promote tissue regeneration rather than its “patching” by scars, which supports the mechanical integrity at the price of reduced function. In this study, we used CS as a feasible model of human MSC spontaneous self-organization to demonstrate its structural, transcriptomic impact and cell commitment, as well as investigated the potential molecular effectors behind the observed phenomena.

## 2. Materials and Methods

### 2.1. Cell Cultures and Reagents

The human adipose tissue-derived telomerase-immortalized MSC cell line (ASC52telo, SCRC-4000™) was purchased from ATCC (Manassas, VA, USA) and cultured in AdvanceStem culture medium (Hyclone, Logan, UT, USA) supplemented with 10% Mesenchymal Stem Cell Growth AdvanceSupplementTM (HyClone, Logan, UT, USA), 1% penicillin/streptomycin solution (HyClone, Logan, UT, USA) and 1% GlutaMAX-1 (Gibco, Waltham, MA, USA).

All cells were cultured at 37 °C and 5% CO_2_; the culture medium was changed every 2 to 3 days. Upon reaching 80%–90% of the monolayer density, the cells were reseeded at a 1:4 ratio using 0.05% Trypsin-EDTA (Gibco, Waltham, MA, USA).

The chemical inhibitor of Rho-associated protein kinase-1 and 2 (ROCK-1/2 or ROCK), Y-27632 ((R)-(+)-trans-4-(1-Aminoethyl)-N-(4-Pyridyl)cyclohexanecarboxamide dihydrochloride), was purchased from Sigma-Aldrich (Y0503, Sigma-Aldrich, Burlington, MA, USA).

The chemical inhibitor of Sterol regulatory element-binding protein (SREBP) truncation and activity, betulin, was purchased from Betulafarm (Betulafarm, Perm, Russia).

Human recombinant transforming growth factor β1 (hTGF-β1 and TGF-β) was purchased from Cell Signaling Technology (8915, Cell Signaling Technology, Danvers, MA, USA).

### 2.2. Cell Sheet Assembly Procedure

To assemble cell sheets (CS), MSC were seeded at a high density ≈ 15,000 cells/cm^2^ (60,000 cells per well in a 12-well CELLSTAR culture plate (Greiner Bio-One GmbH, Kremsmünster, Austria) or 300,000 cells per 60-mm Petri dish (Corning, Corning, NY, USA). During the first 7 days, the cell cultures achieved sufficient density for the assembly of CS, and the formation of condensed and scattered areas within CS occurred by days 10–14; throughout CS assembly, the medium was changed every 2 days. A time-lapse observation of the CS assembly was performed in accordance with the methods in Table 1.

### 2.3. Cell Culture Hematoxylin Staining in Cultures Dishes and Plates

To visualize the outcome of CS assembly and associated MSC self-organization, the cell cultures were stained by hematoxylin. The CS or monolayer cultures at certain time points were fixed with 4% formaldehyde (PanReac AppliChem, Chicago, IL, USA) for 10 min, permeabilized by 70% isopropyl alcohol for 3 min, washed with distilled water, stained with hematoxylin (DAKO, Glostrup, Denmark) for 30 s and then gently washed with tap water for 3 min. Upon the completion of labeling, the cultures were analyzed by microscopy (see Table 1).

### 2.4. Immunofluorescent Labeling of α-Smooth Muscle Actin (α-SMA)

Immunofluorescent labeling of α-SMA was performed directly in culture plates. Prior to labeling, one of the experimental wells was incubated with 5-ng/mL recombinant TGF-β1 (8915, Cell Signaling Technology, Danvers, MA, USA) for 4 days to obtain the positive control culture with prominent α-SMA expression. Then, all the cultures were fixed with 4% formaldehyde (PanReac AppliChem, Chicago, IL, USA) for 10 min, permeabilized for 10 min by 0.02% Triton-X100 (PanReac AppliChem, Chicago, IL, USA) and blocked by 10% goat serum (Thermo-Fisher Scientific, Waltham, MA, USA) in Hank’s Balanced Salt Solution (Paneco, Moscow, Russia) for 1 h. Overnight incubation with the rabbit anti-human antibodies to α-SMA (ab5694, Abcam, Cambridge, UK) was performed at 4 °C. The incubations with goat anti-rabbit AlexaFluor 488-conjugated secondary antibodies (A-11008, Invitrogen, Carlsbad, CA, USA) lasted 2 h at room temperature. The nuclei were labeled with 4′,6-diamidino-2-phenylindole (DAPI; D9542, Sigma-Aldrich, Burlington, MA, USA). After each step, except for blocking, the wells were washed three times with phosphate-buffered saline (PBS). Upon the completion of labeling, PBS remained in the wells, and the samples were analyzed by microscopy (see Table 1). Detailed results of the immunofluorescent detection of α-SMA are presented in Supplementary File “SF-1 aSMA”.

### 2.5. Phalloidin Fluorescent Staining

For F-actin fluorescent labeling, the cells were fixed with 4% formaldehyde (PanReac AppliChem, Chicago, IL, USA) for 10 min and incubated with Alexa Fluor 594 Phalloidin (A12381, Invitrogen, Carlsbad, CA, USA) at 37 °C for 1 h. After each step, the wells were washed three times with PBS. Upon the completion of labeling, PBS remained in the wells, and the samples were analyzed by microscopy (See Table 1).

### 2.6. PicoGreen Dye DNA Assay

To adjust for a different number of cells in the monolayer and CS cultures, as well as in the condensed and scattered areas of CS in microdissected samples, we measured the DNA amount in lysates as previously described using a PicoGreen molecular probe [34]. After the preliminary validation, we established a protocol for MSC to ensure that the amount of DNA in the lysed samples correlated linearly with the cell number in the cultures (Supplementary File “SF-2 PicoGreen validation and calibration”). Thus, we used the DNA amount in the lysates as a surrogate index of the cell number to normalize the values obtained for the other analytes. Briefly, their respective cultures were lysed in 500 μL (per well of a 12-well plate) or 50 μL (per microdissection sample) of ExtractRNA (Eurogen, Moscow, Russia) and disintegrated by pipetting for 10 min on ice. The obtained lysates were diluted (1:50) with TE buffer, and 100 μL of diluted lysates were added to the wells of a 96-well plate and mixed (1:1) with previously prepared Quant-iT PicoGreen dsDNA dye (Thermo-Fisher Scientific, Waltham, MA, USA). The plate was incubated in the dark for 10 min, and the fluorescence emission was measured at 520 nm (excitation at 480 nm) using an EnVision Multilabel Plate Reader (PerkinElmer, Waltham, MA, USA). Each sample was analyzed in duplicate, and bulk measurements were plotted against the data from parallel samples obtained from cultures with known amounts of cells. The resulting calibration curve was used to calculate the DNA amount in assayed samples. Measurements of DNA data are provided in Supplementary Files “SF-3 PicoGreen DNA count” and “SF-4 PicoGreen and LDH microDissection”.

### 2.7. Adipogenic, Osteogenic and Chondrogenic Differentiation of MSC

For the differentiation of the MSC StemPro Osteogenesis Differentiation Kit (Gibco, Waltham, MA, USA), the StemPro Chondrogenesis Differentiation Kit (Gibco, Waltham, MA, USA) and StemPro Adipogenesis Differentiation Kit (Gibco, Waltham, MA, USA) were used according to the manufacturer’s instructions. All the differentiation experiments (including an inhibitor analysis) were carried out in 12-well culture plates after CS assembly, as described above. At Day 12 of CS assembly, the parallel monolayer cultures were seeded using 60,000 MSC per well. Two days later (Day 14 of CS assembly and Day 2 of monolayer culture), the growth media were replaced by the respective differentiation media in experimental wells or by DMEM (Gibco, Waltham, MA, USA) with 10% of FBS (HyClone, Logan, UT, USA) in control wells, and further time points were counted starting from Day 0 of the differentiation. Subsequently, all the media were changed every 3 days. The mineral deposition by osteoblasts was stained at Day 15 of the differentiation with Alizarin red solution (Sigma-Aldrich, Burlington, MA, USA), and lipid droplets in adipocytes were visualized at Day 18 by Oil Red O solution (Sigma-Aldrich, Burlington, MA, USA) staining, according to the manufacturer’s instructions. Chondrogenic differentiation was assessed at Day 21 after staining by Toluidine blue (0.1% solution in 1% sodium chloride, pH 2.3) (Sigma-Aldrich, Burlington, MA, USA). After microscopy and image acquisition (see Table 1), the retaining dyes were extracted from cultures by pure DMSO (PanReac AppliChem, Chicago, IL, USA), and the absorbance at 560 nm (Alizarin Red O), 608 nm (Toluidine blue) or 530 nm (Oil Red O) was measured using an EnVision Multilabel Plate Reader (PerkinElmer, Waltham, MA, USA). To adjust for a different number of cells in the monolayer and CS, the absorbance value reflecting the relative dye amounts in each lysed culture was divided by the amount of DNA obtained for this culture in parallel by the PicoGreen assay described above. A similar value for the respective control culture (not induced to differentiation) reflecting background dye retention was subtracted from the value obtained for the differentiated culture to provide quantitative normalized relative dye retention, reflecting the differentiation efficacy. The detailed normalized data are presented in Supplementary Files “SF-5 DMSO-Elution AlizarinRed normDNA”, “SF-6 DMSO-Elution ToluidineBlue normDNA” and “SF-7 DMSO-Elutions OilRed normDNA”.

### 2.8. Alkaline Phosphatase Activity Assay

To assay the local alkaline phosphatase (ALP) enzymatic activity in CS, we used a BCIP/NBT Substrate Kit (SK-5400, Vector Laboratories, Burlingame, CA, USA). At Day 14 of CS assembly, the culture was washed with PBS, fixed with formaldehyde 4% for 30 s, washed again and permeabilized (Triton X-100 0.1% and Tween-20 0.25%) for 3 min. After washing with PBS, a solution of ALP chromogenic substrate prepared according to the manufacturer’s instructions was added for 10 min. After the completion of staining and washing 3 times with PBS, the images were acquired (see Table 1) and analyzed. A similar procedure was carried out with the MSC monolayer culture and demonstrated an absence of ALP activity within the assay’s limits of detection (data not shown).

### 2.9. MitoTracker™ Probing for Mitochondrial Transmembrane Potential

To evaluate the mitochondrial transmembrane potential (MtMP) in condensed and scattered areas of CS, we used the simultaneous staining of living cells by MitoTracker™ Red CMXRos (red fluorescence) for membrane potential imaging and MitoTracker™ Deep Red FM (far red fluorescence) for the total mitochondria localization (both Thermo-Fisher Scientific, Waltham, MA, USA), according to the manufacturer’s instructions. During the subsequent imaging (see Table 1), the fluorescence intensity ratio obtained from these two probes was used as an accurate indicator for MtMP.

Prior to the MitoTracker™ probe addition, condensed and scattered areas within preassembled CS were manually marked by a permanent marker on the plate’s bottom surface to minimize the exposure during microscopy. A double-staining MitoTracker™ working solution was prepared in HBSS with 100-nM concentrations of both probes. Then, the cultural medium was replaced by a prepared working solution, and the cells were incubated in a CO_2_ incubator for 10 min. Then, the cells were washed with fresh HBSS and incubated in a CO_2_ incubator for 10 min again. After that, previous microphotographs of marked areas of CS were obtained using phase contrast, red and deep red channels (see Table 1). To compare the local MtMP in condensed and scattered areas, two approaches were used: (1) the comparison of red-to-far-red fluorescence intensity ratios and (2) the comparison of the Pearson correlation value for individual pixel brightness in these channels. In both cases, we found no statistically significant differences of the MtMP between condensed and scattered areas. Detailed data is presented in Supplementary Files “SF-8 MtMP”, “SF-9 Mitotracker Ratios” and “SF-10 Mitotracker Pearsons R-value”.

### 2.10. Laser Microdissection Procedure

To separate CS condensed and scattered areas, laser microdissection was used with the Leica laser microdissection system LMD 6000 (Leica Microsystems, Wetzlar, Germany). The general principle was described in Reference [35]. Briefly, MSC were seeded on microdissection dishes with a polyethylene bottom (FWST-5030, WillCo Wells B.V., Amsterdam, The Netherlands). Obtained as previously described, the CS were fixed with 70% ethanol, and the condensed and scattered areas were microdissected under visual control. The dissected areas were collected with a sterile 1.5-mL polypropylene tube (Eppendorf, Hamburg, Germany). The obtained samples (*n* ≈ 50 per tube) were lysed in appropriate buffer and used for downstream applications: Western blotting, solid-phase dot-ELISA, PicoGreen DNA assay, LDH activity assay and RNA isolation for RNA-sequencing.

### 2.11. Protein Isolation, Electrophoresis and Western Blotting

The total proteins were extracted using cell lysis buffer (62.5-mM Tris-HCl (pH 6.8), 7.5% glycerol, 2% SDS, 0.0125% Bromophenol blue and 1.25% β-mercaptoethanol) supplemented with a protease inhibitor cocktail (Roche, Basel, Switzerland) and HaltTM phosphatase inhibitor cocktails (Thermo-Fisher Scientific, Waltham, MA, USA). The extract was cleared by centrifugation (14,000× *g*, 5 min, +4 °C), and the protein concentration was assayed using the Bradford method. Stacking (4% acrylamide) and resolving (12.5% acrylamide) gels were used with an acrylamide-to-methylene-bis-acrylamide ratio of 37.5:1. After the electrophoresis proteins were transferred to a polyvinylidene fluoride (PVDF) membrane (Amersham, Chicago, IL, USA) in a cold room for 16 h at a constant 30 V in Bjerrum and Schafer–Nielsen transfer buffer (48-mM Tris-HCl buffer, pH 9.2, 39-mM glycine and 20% ethanol). After, the transfer membranes were blocked for 1 h by 5% nonfat milk solution in PBST and incubated with solutions containing a primary antibody against the following human antigens: FAK (3285, Cell Signaling Technology, Danvers, MA, USA), pFAK (Tyr397) (3283, Cell Signaling Technology, Danvers, MA, USA), YAP1 (4912, Cell Signaling Technology, Danvers, MA, USA), PYAP1 (Ser127) (4911, Cell Signaling Technology, Danvers, MA, USA), GAPDH (14C10:2118, Cell Signaling Technology, Danvers, MA, USA), COX-IV (4850, Cell Signaling Technology, Danvers, MA, USA), HIF-1α (ab2185, Abcam, Cambridge, UK) and β-actin (ab6276, Abcam, Cambridge, UK). After washing by PBST, the corresponding secondary antibody solutions were added (P-GAM Iss or P-GAR Iss, IMTEK, Moscow, Russia). Detection was performed using Clarity ECL Solution (1705061, Bio-Rad Laboratories, Hercules, CA, USA) or ClarityMax ECL Solution (1705062, Bio-Rad Laboratories, Hercules, CA, USA), followed by visualization in ChemiDoc Touch (Bio-Rad Laboratories, Hercules, CA, USA). A semi-quantitative evaluation of the bands was performed by densitometry ImageLab software (Bio-Rad Laboratories, Hercules, CA, USA). Relative fractions of p-FAK and pYAP1 were determined by normalizing the level of the corresponding non-phosphorylated form, and the housekeeping protein GAPDH was used as the loading control. The relative fraction of COX-IV and HIF-1α were determined by normalizing them to the level of the housekeeping protein (actin-β). See Supplementary Folder “WB” for details and sources.

### 2.12. Solid Phase Dot-ELISA

The quantitative analysis of collagen I and ED-A fibronectin was carried out by the solid-phase dot-immuno-enzyme analysis (dot-ELISA). Lysed culture samples were dispensed in duplicate at 1 μL to a nitrocellulose membrane (Amersham, Chicago, IL, USA) and air-dried. The subsequent immunodetection used a Western blotting protocol with the incubation of membranes with primary antibody solutions against human collagen I (ab34710, Abcam, Cambridge, UK) or ED-A-fibronectin (ab6328, Abcam, Cambridge, UK), followed by their respective secondary antibody solutions (P-GAM Iss or P-GAR Iss, IMTEK, Moscow, Russia). The proteins of interest were visualized using the Clarity Max ECL chemiluminescence kit (Bio-Rad Laboratories, Hercules, CA, USA). Parallel samples with a standard concentration of the protein of interest were made and blotted with a 1:2 step dilution factor and processed as described above to obtain a calibration curve. Quantitative calculations were made in ImageJ (1.53c version, NIH, Bethesda, MD, USA): briefly, the obtained chemiluminescence intensity values were used to obtain the concentrations of the proteins of interest using the respective calibration curves, and the calculated amounts were normalized to the total protein measured using Amido Black (Sigma-Aldrich, Burlington, MA, USA) staining in additional parallel samples [36]. See Supplementary Folder “Dot-ELISA” for the details and measurement data.

### 2.13. Lactate Dehydrogenase Activity Assay

The enzymatic activity of lactate dehydrogenase (LDH) in condensed and scattered areas of CS was assayed in the samples obtained immediately after laser microdissection using a Lactate Dehydrogenase (LDH) Assay Kit (Fluorometric) (ab197000, Abcam, Cambridge, UK) according to the manufacturer’s instructions.

### 2.14. AdipoQ Promoter Reporter Construct and AdipoQRep Cell Line

To generate a feasible model for the visualization of adipogenic differentiation, we used the ASC52telo cell line to establish a reporter line with expression of the GFP gene upon activation of the adiponectin gene (AdipoQ) promoter. A reporter sequence was constructed on the base of the pUCHR-inGFPt lentiviral vector (#60237, Addgene, Watertown, MA, USA). The design of a human adiponectin promoter was based on previously published studies that demonstrated the activity and relative specificity of the minimal murine adiponectin promoter [37,38,39]. The distal (186,840,746,…,186,842,950 bp) and proximal (186,852,825,…,186,853,085 bp) fragments of the adiponectin gene promoter were amplified from human DNA (GCF_000001405.39 (GRCh38.p13)) and cloned to the pUCHR-inGFPt vector upstream of the GFP-coding sequence. The distal fragment included a part of the promoter region, the first exon and a part of the first intron of adiponectin mRNA (−1964…+241 from the start of the transcription initiation). The proximal fragment included the end of the first intron and a part of the second exon of adiponectin mRNA (+10116…+10376 from the start of the transcription initiation). The resulting vector also included a puromycin resistance cassette for the selection of transduced cells. The sequence of the resulting vector was confirmed by Sanger sequencing (see Supplementary Folder “AdipoQRep” for the vector map and sequence). The resulting genetic construct was used to assemble lentiviral particles and transduce ASC52telo cells according to the previously described protocols [40]. Cell selection on puromycin started a week post-transduction and lasted for 2 weeks.

### 2.15. Adiponectin Reporter Assay and ROCK-1/2 Inhibitory Analysis

AdipoQRep were used to obtain the CS and monolayer cultures as described above prior to adipogenic differentiation with an additional control using the CS or monolayer of nonmodified ASC52telo. During this experiment, the local AdipoQ gene promoter activation was visualized in AdipoQRep cells as a GFP signal.

Briefly, the CS and monolayer from AdipoQRep or nonmodified ASC52telo were subjected to adipogenic differentiation for 18 days with a daily time-lapse analysis of green channel fluorescence with unified acquisition settings. An ASC52telo control was introduced to exclude autofluorescence noise in the GFP channel and to control the differentiation ability of AdipoQRep vs. the nonmodified cell line. Local autofluorescence in the GFP channel was used as a threshold for the corresponding AdipoQRep cultures. We found no significant GFP signal in the AdipoQRep cultures without the induction of adipogenesis throughout the experiment time course. At the experiment’s endpoint (Day 18 of the differentiation), all the cultures were stained with DAPI (1:10,000, 15 min) to visualize the nuclei density in the CS areas and monolayer. Different areas of the CS and monolayer culture were acquired using phase contrast (to identify condensed areas) and fluorescent (for GFP and DAPI) microscopy using a Leica DMi8 inverted fluorescence microscope with a DFC7000T camera (Leica Microsystems, Wetzlar, Germany) at unified settings.

The obtained images were processed (see Table 1), and after the trimming of nonspecific signal local levels of GFP and DAPI, the fluorescence was quantified. To do this, we marked the boundaries of the condensed and scattered areas using phase-contrast images. Then, within the marked boundaries, we measured the GFP (green) and DAPI (blue) fluorescence integral densities using the Analyze/Measure tool in the Fiji plugins.

To account for different cellular densities within the CS areas, we divided the local GFP (green) integral density index by the DAPI (blue) integral density index within the same area. The obtained normalized values reflecting the local AdipoQ promoter activity were compared between different cultures (monolayer vs. CS), areas of CS (condensed vs. scattered) and in cultures treated by inhibitors vs. the control. Detailed quantification data is provided in Supplementary File “SF-11 Measurement AdipoQRep vs. DAPI”.

### 2.16. RNA-Seq Sample Preparation and Data Analysis

For total RNA extraction microdissected samples were lysed in Trizol reagent (Sigma-Aldrich, Burlington, MA, USA) according to the manufacturer’s instructions. The RNA sample quality was controlled using BioAnalyser and the RNA 6000 Nano Kit (both Agilent Technologies, Santa Clara, CA, USA). PolyA RNA was purified with the Dynabeads^®®^ mRNA Purification Kit (Ambion, Waltham, MA, USA). An Illumina library was made from polyA RNA with NEBNext^®®^ Ultra™ II RNA Library Prep (New England Biolabs, Ipswich, MA, USA), according to the manufacturer’s instructions. Concentrations of nucleic acids in the obtained libraries were analyzed by the Qubit dsDNA HS Assay Kit using Qbit 2.0 equipment (Thermo Fisher Scientific, Waltham, MA, USA). The distribution of library fragment lengths was assessed using the Agilent High-Sensitivity DNA Kit (Agilent Technologies, Santa Clara, CA, USA). Single-read sequencing with a 50-bp read length was performed on a HiSeq1500 (Illumina, San Diego, CA, USA).

A reads quality check was performed in FastQC [41]. The reads were quality-trimmed (i.e., sequencing adaptors removed) using cutadapt [42], sequence ends with quality scores < 20 were trimmed and sequences < 30 bp long were removed using a sickle [43]. The mapping of trimmed reads to human genome GRCh38 assembly (hg38) and counting the number of reads per gene were performed using STAR [44].

A statistical analysis of the differential expression was conducted using the edgeR package [45]. Raw counts were filtered to leave only the genes that had greater >1 count per million (cpm > 1) in at least two samples (replicates). The remaining read counts were then normalized using the trimmed mean of the values method (TMM) implemented in edgeR to adjust for the variability of the library size. General linear models (GLMs) and the likelihood ratio test (LRT) were used to identify the genes differentially expressed between the conditions in edgeR. Adjustment for multiple comparisons was conducted using the Benjamini-Hochberg false discovery rate (FDR) method (α = 0.05).

The Gene Ontology (GO) enrichment analysis, as well as the Reactome pathway analysis of the differentially expressed genes (DEGs), were implemented using the clusterProfiler [46] and reactome [47] packages. The ggplot2 and gplots libraries were used for graphical representation of the data [48].

### 2.17. Statistical Analysis

A statistical analysis was performed in GraphPad Prism (8.0 version, GraphPad Software, San Diego, CA, USA). The values were expressed as the mean ± SD for normally distributed data and as the median and percentiles (25–75%) for the nonparametric data. Once the normality of the data was confirmed by the Kolmogorov–Smirnov test and Shapiro–Wilk’s W test, we used a Student’s *t*-test, F-test and Tukey’s multiple comparisons test for the comparison of independent groups; the nonparametric values were compared by the Mann–Whitney test, Dunn’s multiple comparisons test or the two-stage linear step-up procedure of Benjamini, Krieger and Yekutieli. Multiple comparisons were performed using one-way ANOVA for normally distributed data and the Kruskall–Wallis test in other cases. The differences were considered statistically significant at *p* < 0.05. In the figure graphs and histograms, significant differences were marked by * (*p* < 0.05), ** (*p* < 0.005), *** (*p* < 0.0005) or **** (*p* < 0.0001).

## 3. Results

### 3.1. MSC Self-Organization in Cell Sheet Occurs through Condensation Mediated by Actin Cytoskeleton Rearrangement

**Figure 1:**
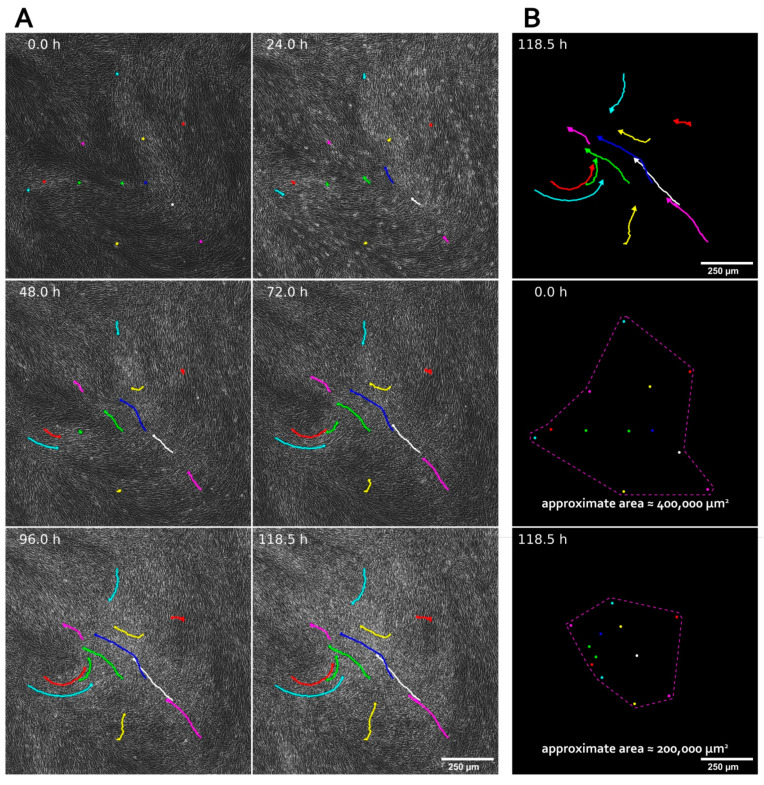
Formation of condensed areas in cell sheets by the spontaneous self-organization of MSC. (**A**) Time-lapse footage of MSC condensation during cell sheet assembly. Phase-contrast microscopy, magnification ×100. Colored dots mark randomly selected cells at the starting point of the observation (0 h), and colored tracks mark their migration during 120 h of experiment. (**B**) Plotted tracks of selected MSC during cell sheet assembly and the analysis of compaction by measurement of the surface area between the most distant selected dots, which decreased approximately 2-fold upon completion of the experiment.

Spontaneous self-organization of MSC was assessed during CS assembly from an initially homogenous dense monolayer of cells. We found that, by days 12–14, MSC formed dense sheets with the spontaneous emergence of numerous compartments with high cell density we termed “condensed areas”. Their formation was characterized by the increased motility of MSC in associated groups ending up in the focal condensation of a cellular mass (Figure 1 and Supplementary Files SF-12, 13, 14 and 15 for the videos). Besides the condensed areas, one may visually identify the regions of CS with a lower MSC density termed “scattered areas”. Generally, our data suggests that the formation of condensed areas resulted in approximately two-fold compactization of the CS surface, and the culture landscape acquired a distinct “hills and valleys” pattern resembling the observations reported for primary smooth muscle cell cultures several decades ago [49,50,51].

**Figure 2:**
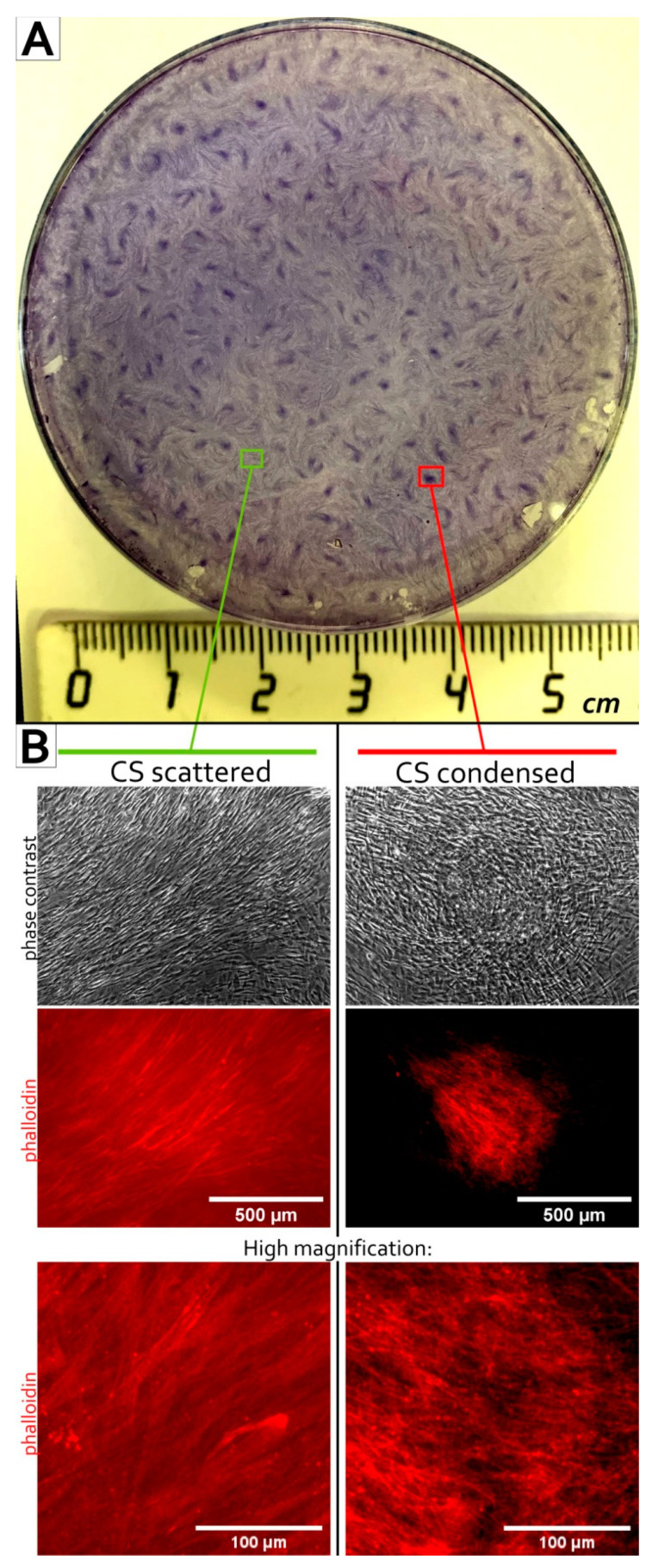
Cytoskeleton of the MSC within condensed areas of cell sheets (CS) is characterized by the high polymerization of F-actin to fibrillar structures. (**A**) Condensed areas are visualized as cell-rich regions with profound staining surrounded by scattered areas characterized by mild staining. CS were assembled from MSC cultured in a 60-mm Petri dish for 14 days; nuclei-stained by hematoxylin; scale bar—5 cm. (**B**). Condensed and scattered areas of CS demonstrate different patterns and the polymerization of the actin cytoskeleton. In condensed areas, sharply delineated F-actin fibers are visible (labeled by fluorophore-conjugated phalloidin), while, in scattered areas, a uniform background is visualized even at increased signal gains; fluorescent and phase-contrast microscopy; magnification ×100 and ×400 in the lower row of images from the scattered and condensed areas.

The active migration of cells (see Supplementary Files SF-12, 13, 14 and 15 for videos) involves an intensive cytoskeleton rearrangement. We used fluorophore-conjugated phalloidin to visualize F-actin fibrillar structures in assembled CS. Condensed areas of CS harbored MSC with prominent F-actin filament polymerization and clearly outlined the fibrils (Figure 2). Scattered areas demonstrated a homogeneous background signal from stained F-actin even at an amplified gain, suggesting a low polymerization of the actin cytoskeleton. This pattern suggested that the self-organization of MSC to condensed aggregates was accompanied by a dramatic change of the cytoskeleton status.

Despite the known in vitro ability of MSC to differentiate into myofibroblasts that may also acquire a high contractility, we found only a scarce number of cells expressing α-smooth muscle actin (α–SMA) in CS (Supplementary File “SF-1 aSMA”).

### 3.2. MSC within CS Readily and More Effectively Undergo Osteogenic and Chondrogenic but Not Adipogenic Differentiation

**Figure 3:**
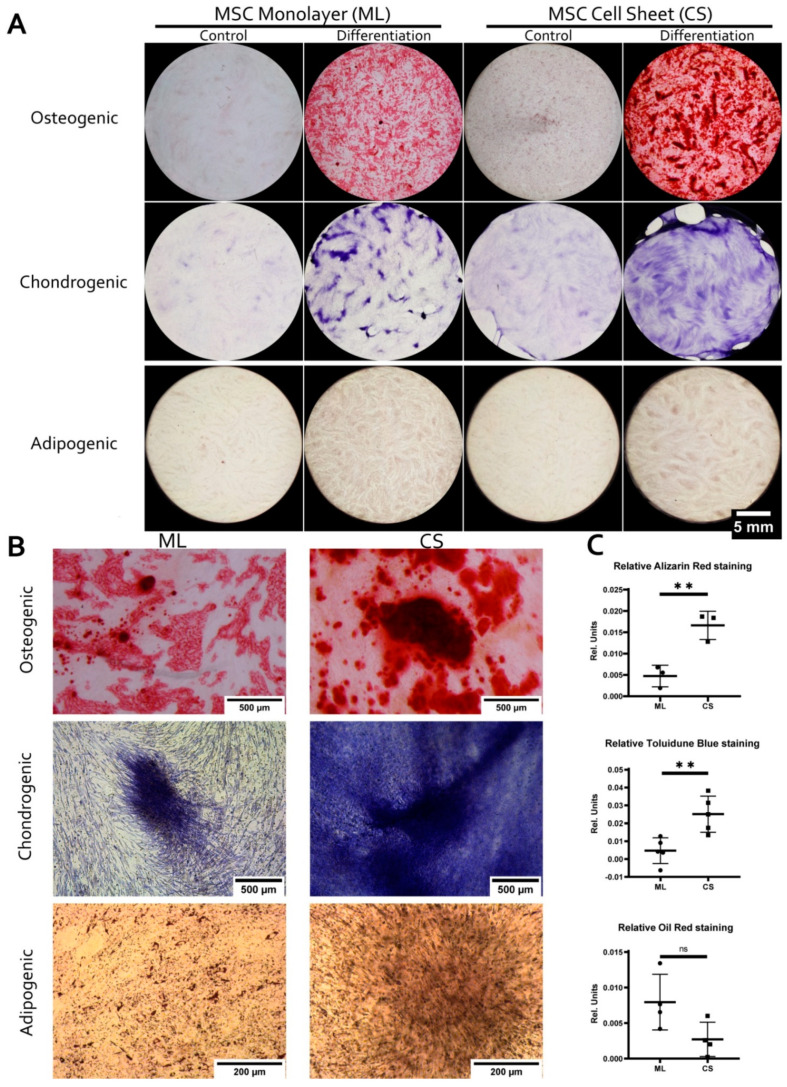
Analysis of MSC differentiation demonstrates a preference for condensation-dependent lineages (osteogenic and chondrogenic) in cell sheets (CS) compared to monolayer cultures. (**A**) MSC monolayer and CS cultures stained after osteo-; chondro- and adipogenic differentiation (15 days for osteogenic, 21 days for chondrogenic and 18 days for adipogenic differentiation). Staining by Alizarin red, Toluidine blue and Oil red O in the experimental endpoint. Macrophotograph with a general overview of a 12-well plate well. (**B**) Same cultures as in (**A**) at a higher magnification; light microscopy (brightfield); magnification ×50 for osteogenic and chondrogenic and ×100 for adipogenic differentiation. (**C**) Comparative analysis of the normalized specific dye retention after the differentiation of MSC in the monolayer or CS. Specific dyes were eluted for a subsequent absorbance measurement; the data was normalized to the DNA amount measured in the respective lysates of the cell cultures. In the figure graphs and histograms, significant differences were marked by ** (*p* < 0.005), and not significant differences were marked by ns (*p* > 0.05).

To investigate the impact of condensation in CS on cell commitment as a distinct feature of self-patterning we compared MSC differentiation in dense monolayer and CS. Three conventional protocols for MSC have been evaluated—namely to bone, cartilage, and adipose tissue (Figure 3). After completion of differentiation and staining we eluted the dye and measured its absorbance to quantify retention correlating with differentiation efficacy. To account for different number of cells in monolayer and CS we lysed these cultures and measured DNA using PicoGreen fluorescent dye. Absorbances of eluted dyes were normalized to corresponding amount of DNA providing relative retention of differentiation-specific dyes (Figure 3C).

We found that compared to monolayer MSC within CS readily and more effectively underwent osteogenic and chondrogenic, but not adipogenic differentiation (Figure 3). By day 15 CS subjected to osteogenic differentiation contained bulky aggregates with profound mineralization that originated from condensed areas and were absent in monolayer culture (Figure 3B). As expected, CS were effectively induced to chondrogenic differentiation with contraction and partial detachment of the construct from the dish. Interestingly, during chondrogenic differentiation of monolayer MSC we observed emergence of aggregates with rich Toluidine blue staining resembling condensed areas in CS. At experiment endpoint (Day 21) relative retention of Toluidine blue was significantly higher in CS compared to monolayer (Figure 3C). Both findings were in line with established importance of condensation for solid connective tissue formation [26,52]. To our surprise retention of Oil Red O which reflected efficacy of adipogenesis did not differ significantly (*p* = 0.06) between CS and monolayer cultures at Day 18 of adipogenic differentiation (Figure 3C).

### 3.3. MSC in Condensed Areas Demonstrate Early Signs of Commitment to Osteocytes Occurs Prior to the Induction of Differentiation

We noted that during osteogenic differentiation of CS its condensed areas had the most significant morphological changes of cells and abundant Alizarin red staining. Thus, enhanced osteogenic differentiation of CS was predominantly confined to condensed areas which suggested analysis of osteogenic markers emergence prior to induction of differentiation.

**Figure 4:**
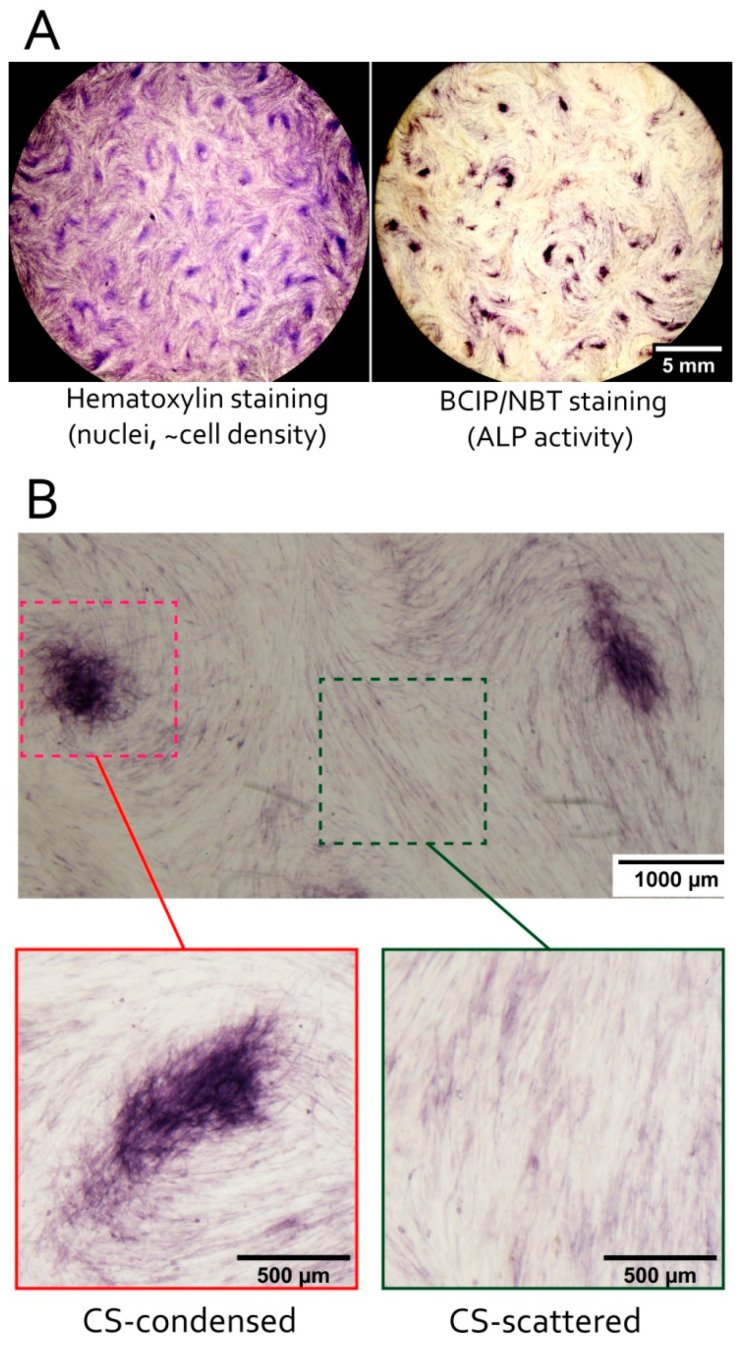
The local activity of alkaline phosphatase (ALP) spontaneously increases in MSC within condensed areas of the cell sheet (CS). (**A**) MSC sheets stained by hematoxylin to visualize the cell density (left) or by BCIP/NBT chromogenic substrates to visualize the ALP activity (right). We present an image of a CS cultured in parallel with one subject to ALP visualization; light microscopy (brightfield); scale bar 5 mm. (**B**). Representative images of condensed areas with obvious ALP activity visualized after treatment by specific chromogenic substrates; light microscopy (brightfield); magnification 40× (upper image) and 80× (lower row of images).

Alkaline phosphatase (ALP) is often used to evaluate the balance of commitment and stemness in MSC and other adult stem cells [53]. Upregulation of ALP in MSC is also a sign of osteogenic events and early stages of MSC differentiation to osteoblasts. Pre-formed undifferentiated CS were treated by a chromogenic substrate reacting with ALP to form insoluble dark blue or purple precipitates. We found higher enzymatic activity of ALP within condensed areas compared to scattered areas of CS (Figure 4). It is unlikely that increased ALP activity in condensed areas was observed due to higher cell number since scattered areas had marginally no ALP activity despite presence of MSC visualized by parallel hematoxylin staining. Thus, ALP activity was predominantly confined to condensed areas of CS (Figure 4).

**Figure 5:**
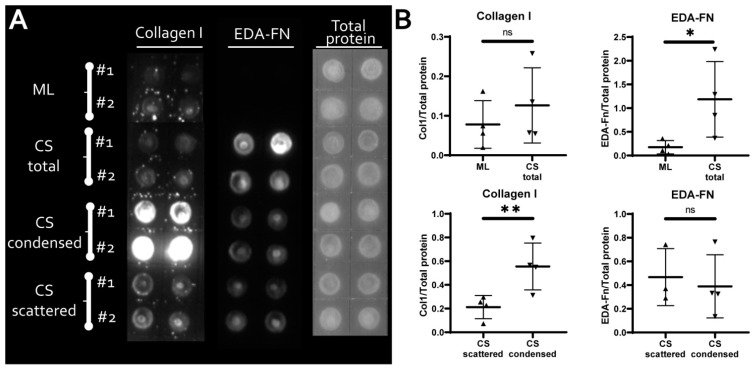
Characterization of the ECM composition in the MSC monolayer and cell sheets (CS) demonstrates the differential deposition of collagen-I and EDA-fibronectin. (**A**) Representative images of the collagen-I and EDA-fibronectin assays in lysates of a monolayer culture, total CS and microdissected condensed and scattered areas using dot-ELISA. One of the 4 biological replicates is presented. (**B**) Statistical analysis (*n* = 4) of the ECM components (collagen-I and EDA-fibronectin) content normalized to the concentration of total protein measured by Amido black (panel **A**, right pair of lanes). In the figure graphs and histograms, significant differences were marked by * (*p* < 0.05), ** (*p* < 0.005), and not significant differences were marked by ns (*p* > 0.05).

To analyze ECM composition which may influence the efficacy of osteogenic differentiation major matrix ECM proteins collagen I and ED A-fibronectin were assayed using solid-phase dot-ELISA in monolayer and CS cultures as well as in condensed and scattered areas microdissected from CS (Figure 5). Relative content of assayed proteins in lysates has been obtained by data normalization to total protein concentration measured by amido black stain in parallel. In lysates of CS, the collagen I content has been similar to monolayer while ED A-fibronectin showed an approx. five-fold increase CS compared to the monolayer (Figure 5, upper part). To further strengthen this observation, two mechanosensing signaling systems activity were assessed using western blotting: focal adhesion kinase (FAK) and Yes-associated protein 1 (YAP). We found that CS lysates showed higher content of active phosphorylated (Tyr397) focal adhesion kinase (pFAK) compared to monolayer MSC; both were normalized to total FAK within each sample (Supplementary File “SF-16 FAK/YAP”). Analysis of YAP/TAZ pathway activation using YAP1 as a marker signaling molecule demonstrated that inactive phosphorylated YAP1 (pYAP1) was increased in CS compared to monolayer MSC (Supplementary File “SF-16 FAK/YAP”). YAP/TAZ is activated by stiff environment or surface and our data shows that MSC indeed perceive CS enriched by ED A-fibronectin as a softer microenvironment compared to monolayer. In microdissected condensed areas, we found enrichment by collagen I with its relative content approx. four-fold higher vs. scattered areas while ED A-fibronectin demonstrated a ≈1:1 distribution (Figure 5, lower part). Collagen-1 is the major component of bone ECM known to promote osteogenic differentiation of MSC and upregulate ALP expression via integrin-mediated activation of ERK1/2 pathway [54,55].

Cell’s stemness/differentiation and metabolism are mutually connected, and it is generally acknowledged that naïve stem cells are characterized by lower metabolic potential with reduced input of oxidative phosphorylation (OxPhos). In contrast, differentiation is accompanied by an increase of OxPhos systems activity. We screened CS cultures for major indicators of cell metabolism:

- mitochondrial transmembrane potential (MtMP);
- content of cytochrome C oxidase complex IV (COX-IV)—the terminal enzyme complex component of the respiratory chain;
- hypoxia inducible factor (HIF-1a);
- activity of lactate dehydrogenase (LDH)—the terminal enzyme of anaerobic glycolysis).

**Figure 6:**
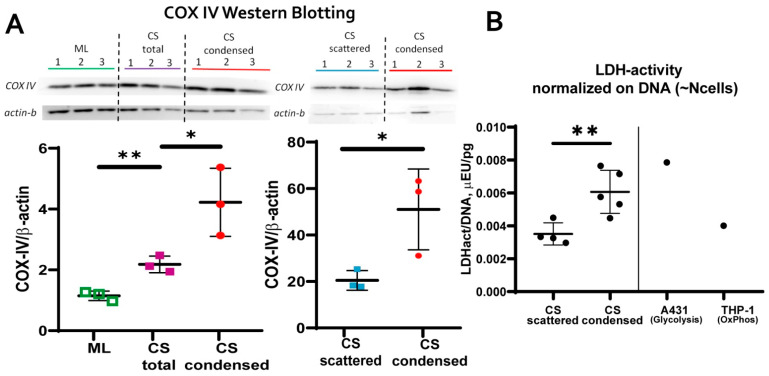
Cells from condensed areas of the cell sheet (CS) are characterized by an increased content of the cytochrome-C oxidase IV component (COX-IV) and higher lactate dehydrogenase (LDH) activity. (**A**) Western blotting of the mitochondrial ETC COX-IV subunit in lysates of ML, total CS and microdissected condensed or scattered areas. Graphs present a densitometry analysis of the COX-IV content normalized to β-actin (densitometry analysis). (**B**) LDH activity (LDHact) in the lysates of microdissected condensed areas and scattered areas. DNA amounts in the respective cell lysates were used for the normalization of LDH activity to the cell number. Control data was obtained from lysates of the A431 cell line that increased the proportion of anaerobic glycolysis and the THP-1 cell line with the major input of oxidative phosphorylation but not glycolysis. The band numbers indicate individual biological replicate samples. In the figure graphs and histograms, significant differences were marked by * (*p* < 0.05) and ** (*p* < 0.005).

Using potential-dependent and independent Mitotracker™ probes or western blotting we found no significant differences between condensed and scattered areas of MtMP (Supplementary File “SF-8 MtMP”) or HIF-1α stability, respectively (Supplementary File “SF-17 HIF1a). However, condensed areas demonstrated increased content of COX-IV (Figure 6A) suggesting higher OxPhos activity [56]. Lactate dehydrogenase (LDH) activity was also increased in condensed areas possibly due to higher metabolite flux to anaerobic glycolysis (Figure 6B), which could be due to higher metabolite flux to anaerobic glycolysis. Interestingly, an increased intercellular LDH/DNA ratio has been demonstrated for osteogenically differentiating MSC [57]. Thus, cells from condensed areas had increased metabolic activity reflecting putative reduction of stemness and early stages of commitment.

We assume that features of condensed areas (increased activity of ALP, enrichment by collagen I and enhanced metabolism) altogether indicate decreased stemness and commitment of MSC harbored in these areas to a direction favoring osteogenic differentiation.

### 3.4. Transcriptomic Analysis of MSC in Scattered and Condensed Areas Reveals Dramatic Changes in Expression Profiles

**Figure 7:**
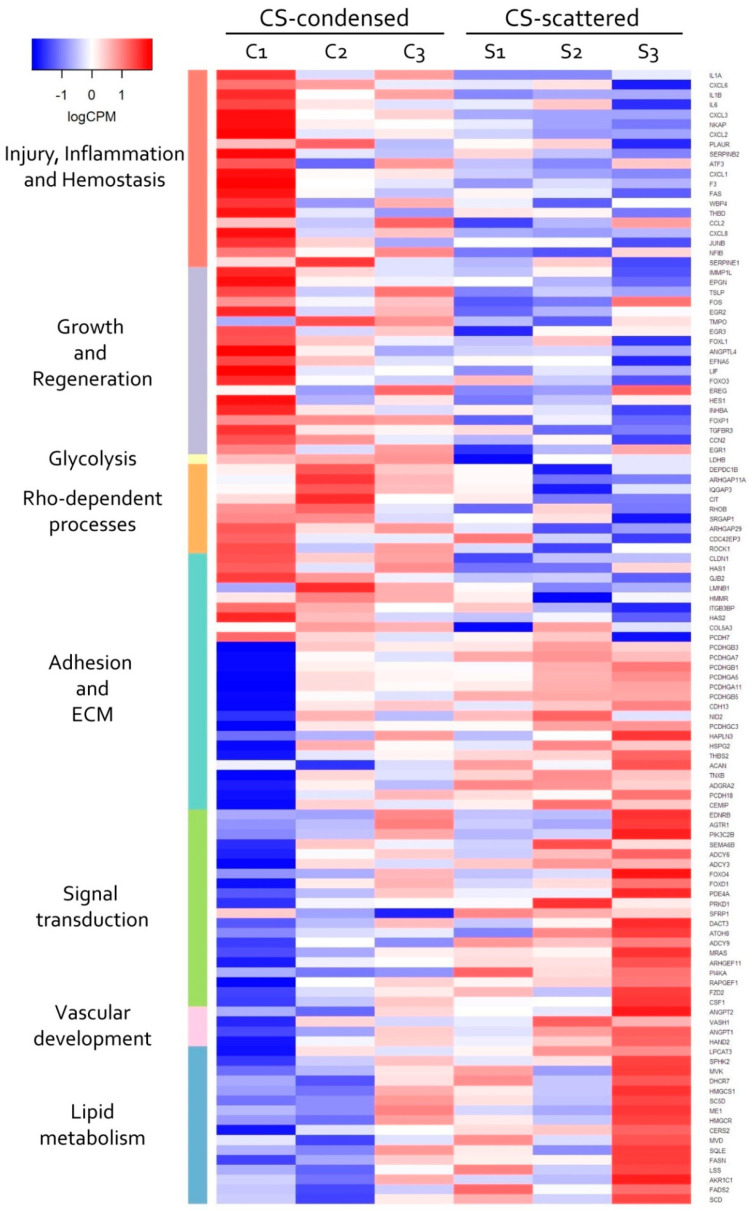
Heat map illustrating differential gene expression between the cells of CS-condensed and CS-scattered areas according to RNA-Seq. The expression values are normalized using CPM (count per million) measures. The columns represent samples, and rows represent upregulated (red) and downregulated (blue) genes. The samples are grouped by conditions and biological replicates. The left colored panel shows the association of the genes with some of the top GO terms. The displayed genes have been manually selected from all DEGs as those representative of the highlighted associated groups.

The observed changes of differentiation and commitment of MSC were expected to involve modulation of transcription factors activity. To test this hypothesis, we screened transcriptomic data for differential gene expression that could underlie the observed phenomena. Comparative analysis of gene-coding mRNAs in condensed and scattered areas of CS required use microdissection of pre-formed construct as described. Dissected samples were lysed and subject to total RNA extraction prior to RNA-sequencing. Obtained library was used for bioinformatic processing a list of differentially expressed genes (DEGs) was obtained. In condensed vs. scattered areas of CS a total of 368 significantly upregulated and 168 downregulated protein-coding DEGs were identified (Supplementary file “SF-18 DE_Genes”). The annotation of these DEGs with Gene Ontology (GO), Reactome pathways and Gene Set Enrichment Analysis (GSEA) algorithms (Supplementary Files “SF-19 GO and Reactome” and “SF-20 GSEA”) demonstrated that the majority of DEGs were assigned to terms with similar or overlapping function(s) involved in cellular processes crucial for tissue formation and repair (Figure 7).

**Figure 8:**
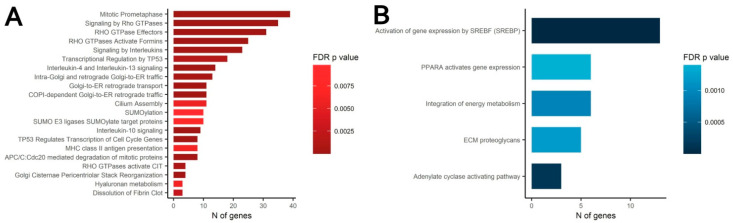
Reactome pathway analysis between the cells of CS-condensed and CS-scattered areas. (**A**) The bar plot shows the number of upregulated genes associated with the top Reactome pathways and the adjusted *p*-values for these pathways (assigned by color). Plot displays the top pathways by the number of genes related to the Reactome pathway (total number of significant genes (ntotal) is 239). (**B**) The number of downregulated genes associated with the top Reactome pathways (ntotal = 112).

Analysis of the GO and Reactome pathways showed that among the downregulated genes a significant part (approx. 8%) were regulators or targets of the steroid responsive-binding element (SREBP) transcription factor (Figure 8B). SREBP is one of the main transcriptional regulators of genes that control lipid synthesis and accumulation. Considering this minor lipid deposition during the adipogenic differentiation of MSC in condensed areas gains a putative explanation. However, among the annotated DEG clusters, we failed to identify the ones associated with MSC osteogenic or chondrogenic differentiation.

However, the Reactome pathways analysis showed that, among the upregulated DEGs, a considerable fraction (approx. 10%) were functionally related to the activity of Rho small GTPases family (Figure 8A). Activity of Rho GTPase is strongly associated with rearrangement of cytoskeleton during migration, division and mesenchymal condensation [58]. Furthermore, activation of the Rho GTPase pathway in MSC is known to induce osteogenic and chondrogenic differentiation of MSC [59,60,61].

Results of bioinformatic analysis that highlighted Rho GTPase pathway were concordant with data on actin cytoskeleton status in condensed areas (Figure 2) and suggested a putative molecular mechanism of MSC condensation and self-patterning during CS formation.

### 3.5. MSC Condensation in Cell Sheets Is Mediated by Activity of Rho GTPase Pathway

RNA-sequencing data suggested that MSC condensation within CS was associated with activation of the Rho GTPase pathway. To validate this finding, we used Y-27632—a specific inhibitor of Rho-dependent protein kinases 1 and 2 (ROCK-1/2)—main downstream molecules of the Rho GTPase signaling pathway.

**Figure 9:**
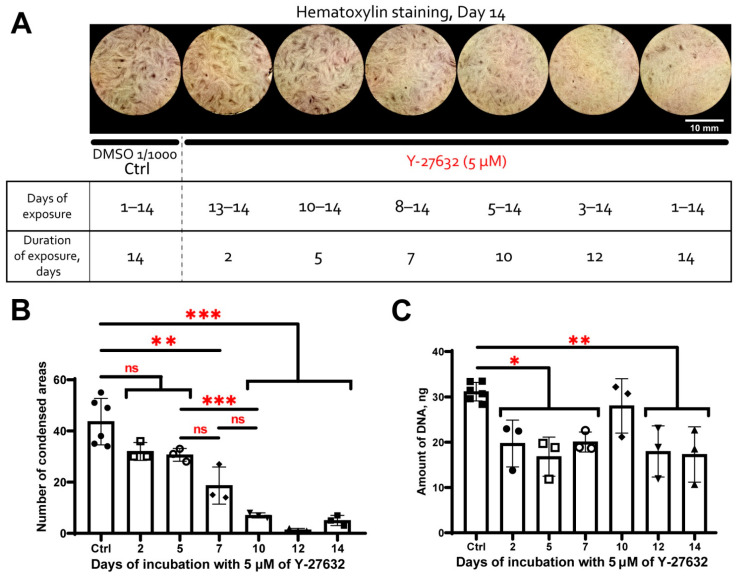
MSC condensation is disrupted by suppression of the Rho/ROCK-1/2 signaling pathway. (**A**) Cell sheet general view at Day 14 of incubation with Y-27635. Hematoxylin staining, macro photographs of a 12-well plate well. (**B**) Comparative analysis of the condensed areas number after the inhibition of Rho/ROCK-1/2 of different durations. Condensed areas number by Day 14 is reduced by the ROCK-1/2 inhibitor (Y-27632) at the early stages of CS formation (Days 0–7). (**C**) Comparative analysis of the DNA amount measured in the respective (**A**,**B**) cell culture lysates after Rho/ROCK-1/2 inhibition. The proliferation of MSC is impaired by Y-27632, but no significant difference between the experimental time points was demonstrated. ROCK1/2-dependent suppression of cellular proliferation has a constant exposure-independent profile and provides a uniform background. In the figure graphs and histograms, significant differences were marked by * (*p* < 0.05), ** (*p* < 0.005) or *** (*p* < 0.0005) and not significant differences were marked by ns (*p* > 0.05).

Y-27632 was added to MSC culture medium at different time points during CS formation. Addition of Y-27632 at 5 μM for 7, 10, 12 and 14 days resulted in significant drop of condensed areas number formed by Day 14 (vs. DMSO) while incubation for 2 or 5 days did not render a statistically significant impact (Figure 9A,B). This suggested that the formation of condensed areas was ROCK-1/2-dependent, and the first 7 days of CS assembly were the pivotal period. Indeed, the addition of Y-27632 at later time points showed marginally no effect on MSC condensation (Figure 9). Hence, Rho GTPase pathway activity and ROCK-1/2 are required to induce and drive MSC condensation but seem dispensable for the support of condensed areas after condensation is completed.

Analysis of the DNA content showed that treatment by Y-27632 resulted in its statistically significant reduction vs. control (DMSO) indicating reduced proliferation due to inhibition of ROCK-1/2 (Figure 9C). All experimental time points (excluding 10 Days) with Y-27632 treatment showed comparable reduction of DNA relative content vs. control and did not differ between each other significantly (Figure 9C). Incubation for 10 days with Y-27632 did not differ statistically vs. DMSO or any other experimental time point. Thus, the reduction of the DNA content was uniform and did not depend on duration of exposure to Y-27632 (Figure 9C). We assume this illustrates rapid adaptation of cell cycle control systems to inhibition of Rho GTPase pathway, as well as the availability of bypass ROCK1/2-independent cascades that support cell proliferation. Despite the suppression of proliferation by Y-27632 cannot be excluded in this assay, reduced MSC condensation after treatment by ROCK-1/2 inhibitor was a specific time-dependent response to abolishment of Rho-GTPase pathway activity indicating its crucial for migration and condensation of MSC.

### 3.6. Reduced Adipogenic Potential of MSC in Condensed Areas Is Mediated by Local Activation of Rho GTPase Pathway

Besides activation of Rho GTPase pathway targets transcriptomic analysis also indicated downregulation of SREBP-dependent mRNAs in condensed areas which was concordant with similar efficacy of adipogenesis in monolayer and CS (Figure 3). Rho-GTPase pathway and efficacy of adipogenesis are connected via regulation of SREBP and one of its crucial targets—fatty acid synthase (FASN) responsible for lipid synthesis and accumulation [62,63,64].

**Figure 10:**
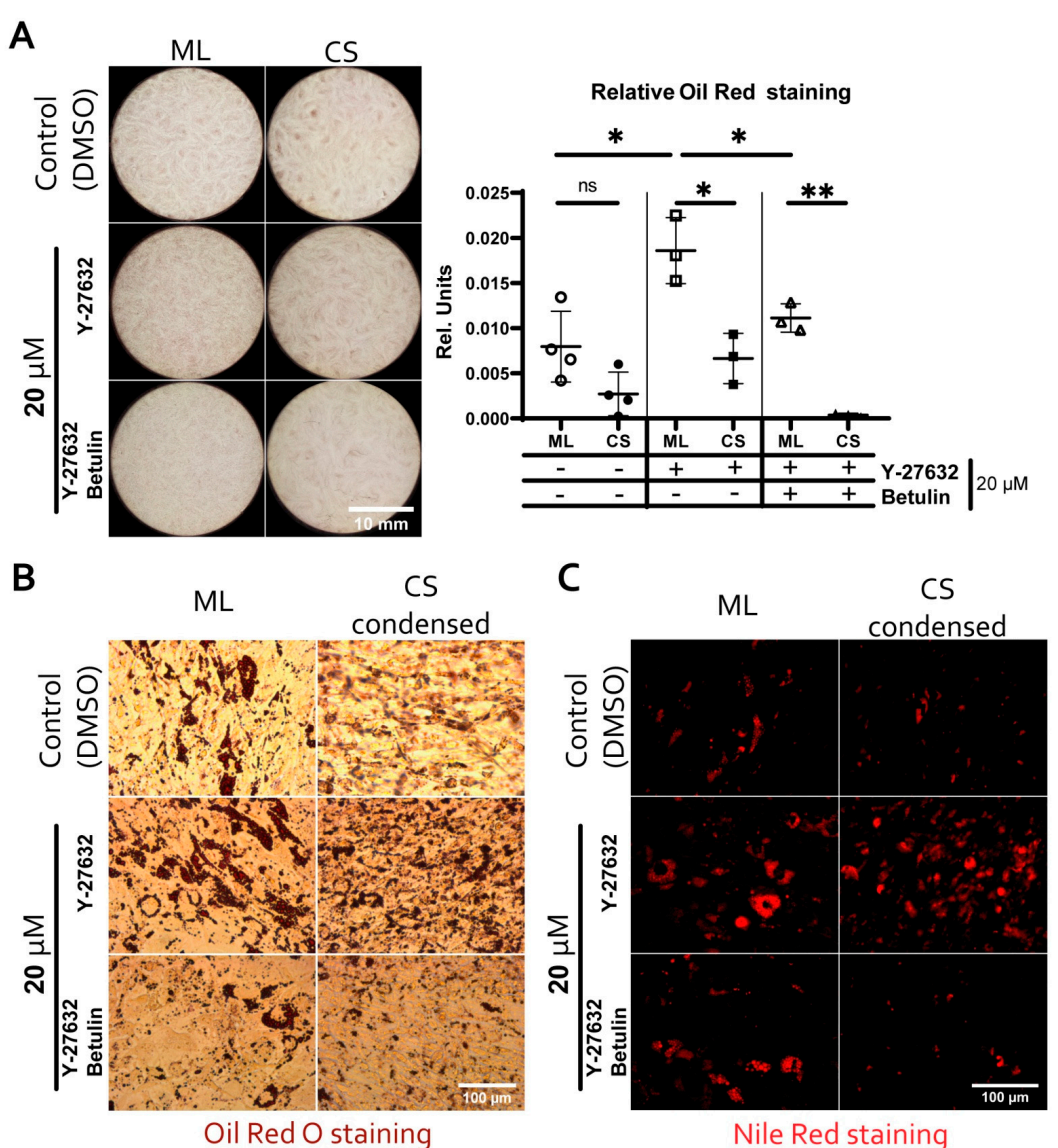
Lipid droplet accumulation during adipogenic differentiation is enhanced by the suppression of ROCK-1/2 pathway activity in the MSC monolayer culture but not in the total cell sheet (CS). Additional inhibition of SREBP abolishes this effect and suppresses lipid deposition in both the monolayer and whole CS. Lipid deposition in condensed areas, as well as in the monolayer, is enhanced by the suppression of ROCK-1/2 activity (Y-27632), while the inhibition of SREBP (betulin) abolishes this effect, reducing the lipid accumulation to a minimum. (**A**) MSC monolayer and CS overview at Day 18 of adipogenic differentiation with or without the addition of Y-27635 and betulin; Oil red O staining. Comparative analysis (right) of normalized Oil red O retention in lysates of cell cultures after differentiation. Oil Red O was eluted for the subsequent absorbance measurement; data was normalized to the DNA amount measured in the respective lysates of the cell cultures. (**B**) Higher magnification images of lipid droplets stained by Oil Red O in the monolayer and CS-condensed areas from the experiment in panel A (**A**,**C**). Light microscopy (brightfield), magnification 50×. (**C**). Lipid droplets stained by Nile Red in the monolayer and condensed areas under the experimental conditions similar to panels A and B; fluorescent microscopy, magnification 50×. See Supplementary Folder “AdipoFigures” for more high-resolution FOWs images. In the figure graphs and histograms, significant differences were marked by * (*p* < 0.05) or ** (*p* < 0.005).

To validate our assumption, we used inhibitors of ROCK-1/2 (Y-27632) and SREBP (betulin) [65] in monolayer and CS cultures undergoing adipogenic differentiation (Figure 10, see supplementary folder “AdipoFigures” for more high-resolution FOWs images). We found that in monolayer, inhibition of ROCK-1/2 resulted in a statistically significant increase of Oil Red O retention indicating higher accumulation of lipids (Figure 10A). Additional inhibition of SREBP by betulin abolished Y-27632-induced increase of lipid accumulation in monolayer MSC dropping it to values of DMSO control (Figure 10A).

Using CS in a similar experimental setting we failed to reproduce above mentioned finding made in monolayer MSC (Figure 10A). We assume it was due to inevitable averaging of data within CS lysate which contained both condensed and scattered areas contributing unequally to overall Oil red O retention values. This hinted at the use of a local analysis of lipid deposition in different CS areas, so we stained the differentiated construct with lypophillic NileRed fluorescent dye and performed a microscopic analysis.

We found that lipid deposition in condensed areas showed a relative increment upon inhibition of ROCK-1/2 (Y-27632), and this effect was abolished by simultaneous inhibition of both ROCK-1/2 and SREBP (betulin) (Figure 10B,C). This result in local CS compartments was concordant with our findings in monolayer culture (Figure 10). Interestingly, in all fields of view (FOVs) size of lipid droplets in terminally differentiated monolayer culture was larger than in MSC within differentiated CS. Thus, in condensed areas, a reduced activity of SREBP was caused by the locally activated Rho-ROCK-1/2-dependent signaling pathway.

### 3.7. During Adipogenic Differentiation Adiponectin (AdipoQ) Expression in Condensed Areas Is Driven by Activation of Rho GTPase Pathway

Using endpoint fluorescent staining to study local accumulation of lipid droplets (Figure 10), we were unable to yield a quantitative comparison of condensed and scattered areas. For this comparative analysis of local adipogenic differentiation, we used a lentiviral vector to create a reporter MSC line designated “AdipoQRep” bearing GFP controlled by the human adiponectin gene (AdipoQ) promoter. Adiponectin expression is confined to adipose tissue exclusively and is known to increase during differentiation of MSC to adipocytes [66].

Basing on this, we assumed that expression of adiponectin would mark the process of adipogenesis in MSC. We assembled CS from AdipoQRep and induced their adipogenic differentiation with or without the addition of ROCK-1/2 and SREBP inhibitors as described above. GFP signal was analyzed using fluorescent microscopy throughout differentiation process (total of 18 days).

**Figure 11:**
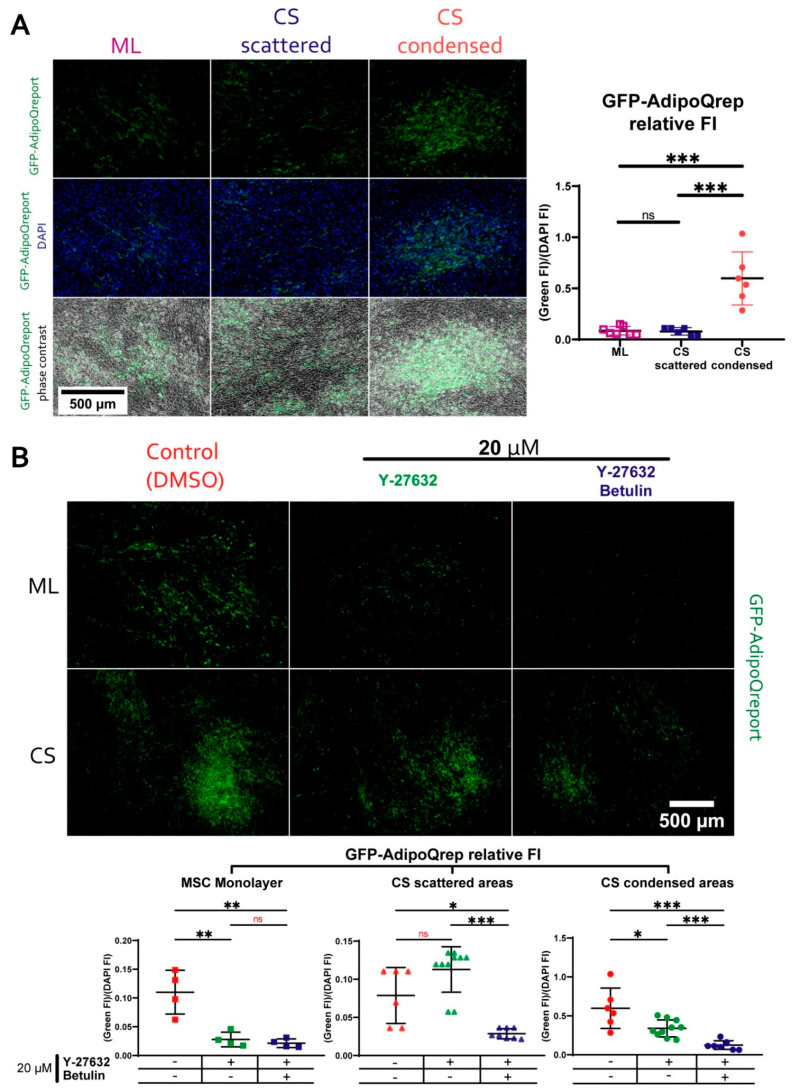
Adiponectin (AdipoQ) gene expression during adipogenic differentiation is differentially mediated by the Rho/ROCK-1/2 pathway in the monolayer culture and cell sheet (CS). Adiponectin promoter bearing genes expression in MSC in the monolayer and CS-condensed and scattered areas have different dependence on the ROCK pathways inhibition during adipogenic differentiation. To evaluate the expression of the AdipoQ gene, we obtained a genetically modified MSC line (AdipoQRep) bearing GFP controlled by the AdipoQ promoter. (**A**) During adipogenic differentiation, the AdipoQ-dependent GFP signal is increased in condensed areas compared to the monolayer (ML) and scattered areas. Ratio of GFP signal reflecting the activation of the AdipoQ gene promoter and DAPI signal was used as a relative indicator of adiponectin expression; fluorescence microscopy, magnification 100×. (**B**) Inhibition of ROCK-1/2 with Y-27632 results in a dramatic drop of the AdipoQ-dependent GFP signal in both ML and condensed areas but not in scattered areas of CS. Addition of betulin suppresses AdipoQ-dependent GFP expression in all experimental settings (compared to the control; fluorescence microscopy, magnification 50×. In the figure graphs and histograms, significant differences were marked by * (*p* < 0.05), ** (*p* < 0.005) or *** (*p* < 0.0005) and not significant differences were marked by ns (*p* > 0.05).

Control samples AdipoQRep CS (without induction of differentiation) demonstrated no GFP signal throughout observation period excluding “leak” of inserted AdipoQ promoter activation (Figure 11). In differentiated CS, we found that in condensed areas signal from AdipoQ-controlled GFP was significantly higher than in both monolayer culture or scattered areas of CS (Figure 11A). Suppression of ROCK-1/2 activity (Y-27632) and SREBP (betulin) significantly reduced AdipoQ-dependent GFP expression in the monolayer and condensed areas by Day 18 (Figure 11B). Y-27632 seemed to render significant effect only in condensed areas of CS compared to scattered areas which failed to respond to Y-27632. Indeed, in scattered areas of CS, Y-27632 failed to disturb AdipoQ expression, while Y-27632+betulin caused a dramatic drop of GFP-signal. Thus, scattered areas seem are likely to have minimal Rho-GTPase pathway activity, while condensed areas differ dramatically, which was concordant with the RNA-seq data (Reactome pathways data in Figure 8). Overall, the expression of AdipoQ was ROCK1/2-dependent, and our data demonstrated that Rho-GTPase pathway was not a suppressor but an activator of AdipoQ, which contradicted our initial assumption.

## 4. Discussion

We investigated the phenomenon of self-organization during assembly of cell sheet (CS) from human adult adipose tissue MSC highlighting its influence on cell fate and differentiation in vitro. Our work also provides an insight to potential mechanisms that mediate self-organization of MSC, their spontaneous condensation and commitment to preferential lineage to bone and cartilage, but not adipose tissue. Ex vivo aggregates of MSC (e.g., spheroids) have been extensively studied as model systems [23,67,68] or therapeutic product [69,70]. However, to our knowledge we are the first to report and address this phenomenon, its transcriptional and cell fate impact in CS—a widely used tissue engineering construct. Generally, we have undertaken an attempt to reconcile our understanding of adult MSC self-organization, its role in morphogenesis in adult body (including regeneration and reparation) with conventional knowledge about mesenchyme condensation.

Discussing the relevance of CS as a model system for studied subject we may indicate their advantages over abovementioned spheroids as an object to study self-organization. Open surface of CS allows active group motion of adherent cells (Figure 1) which is crucial for self-assembly and self-morphogenesis [10].

Within CS intrinsic self-patterning program of MSC has a freedom to unfold and dominate over external inputs that orchestrate cell behavior in spheroid and organoid cultures. Indeed, in spheroids environmental conditions (oxygen and metabolite availability, soluble factors diffusion) drastically differ along the center-periphery axis [71]. In contract, CS provides a relatively homogeneous distribution of signals and cues from the environment especially during early stages of assembly. Thus, anisotropy of cell density and differentiation efficacy we observed after formation of CS primarily originates from emerging spatial heterogeneity of cellular interactions and local rules that control cell position and fate. Finally, anisotropy and topographic relationships between cells are easier to visualize and observe in CS. Within CS we found vivid spontaneous aggregation and contraction of MSC forming two distinct compartments with different cell morphology—namely condensed and scattered areas (Figure 1 and Figure 2).

The main mechanism of embryonic mesenchymal condensation in vivo is actin cytoskeleton polymerization [72]. Within CS condensed areas MSC demonstrated prominent F-actin fibers MSC suggesting actin cytoskeleton rearrangement as a major mechanism of observed cell mass compaction (Figure 2). The similarity of these fibers to α-SMA stress fibrils hinted to exclude spontaneous transition of MSC to myofibroblasts that possess strong contractile ability important during wound closure and healing. However, α-SMA-positive cells (Supplementary File “SF-1 aSMA) were marginally absent in CS indicating that condensation did not involve significant myofibroblast emergence and subsequent α-SMA-dependent contraction.

To study the MSC fate after self-organization, we induced their differentiation to bone, cartilage or adipose tissue in monolayer culture and CS (Figure 3C). Our data suggests that in CS MSC have increased differentiation potential for osteogenic and chondrogenic, but not adipogenic lineage. This finding was well-corroborated by importance of condensed status for bone and cartilage morphogenesis in vivo [73,74,75,76,77,78,79].

Comparable efficacy of adipogenic differentiation in CS and monolayer was an important point indicating that enhanced osteogenic and chondrogenic differentiation was not a simple consequence of higher cell count in CS. We drew a conclusion that in CS intrinsic mechanisms and self-patterning provide preferential directions of differentiation for MSC excluding one of them which complies to description of local environment-dependent commitment [80].

Detailed microscopic evaluation of CS during osteogenic differentiation showed that MSC in condensed areas acquired higher ability for mineral deposition characteristic for bone tissue (Figure 3B). After osteogenic differentiation CS condensed areas were the origins of solid bulky aggregates with prominent Alizarin red staining indicating high content of mineralized matter. This pro-osteogenic status of MSC in condensed areas was supported by spontaneous (before induction of differentiation) increase in local activity of alkaline phosphatase (ALP) (Figure 4) and cells’ metabolic status (Figure 6).

These local alterations reflect a set of conditions that favor osteogenic differentiation of MSC prior to induction of one. ALP activity is essential for ECM mineralization during osteogenesis and considered a valid marker of osteogenic commitment [81]. Increased level of COX-IV in condensed areas (Figure 6A) indicated higher throughput of mitochondrial ETC and/or higher mitochondria number. This piece of evidence indicated greater input of oxidative mitochondrial phosphorylation and overall enhancement of metabolic activity [56,82]. Oxidative phosphorylation and mitogenesis accompany enhanced differentiation of MSC [83], especially, to osteogenic lineage [84]. Finally, observed increase of lactate dehydrogenase (LDH) enzymatic activity (Figure 6B) reflected active aerobic glycolysis characteristic for osteoblasts where its high turnover provides crucial anabolic precursors [85]. For example, during Wnt-induced differentiation of osteoblasts lactate dehydrogenase A (LDHA) upregulation is mandatory and occurs to enhance aerobic glycolysis [86,87,88]. Lactate dehydrogenase B (LDHB) may be involved in conversion of extracellular lactate to pyruvate to supply high mitochondrial demand in differentiating cells with increased ALP activity [89]. Since our analysis of total LDH enzymatic activity does not distinguish between specific contributions by LDHA and LDHB it may require additional study to clarify molecular effectors.

Discussing putative cause of established metabolic profile in MSC we may exclude local hypoxia or metabolic deprivation in condensed areas as far as we found no difference of HIF-1α or mitochondrial membrane potential (MtMP) between condensed and scattered areas (data not shown, see supplementary files “SF-8 MtMP”, “SF-17 HIF1a”). Thus, observed shifts of metabolism are likely to occur due to emerging regulatory circuits between cells, but not by external influences (e.g., lack of oxygen or metabolites).

Another important structural feature of condensed areas that may promote osteogenesis prior to induction of osteogenic differentiation was local enrichment by collagen I—the major component of bone ECM (Figure 5, lower part). MSC committed to osteoblasts increase deposition of collagen I [90] and the latter promotes osteogenic differentiation inducing ALP expression via ERK1/2 pathway [54,91]. Observed formation of bulky mineralized aggregates at differentiation endpoint (Figure 3B) is also in line with these findings as far as collagen I is the main mineralization substrate at terminal stages of bone formation [92]. Specificity of collagen I deposition pattern was supported by contrastingly homogenous distribution of ED A-fibronectin—another ECM protein produced by MSC (Figure 5, lower part). Its relative content did not differ between condensed and scattered areas of CS indicating that deposition of ECM was not correlated to cell number, but was a feature of microenvironments within CS depending on local rules and self-patterning.

Thus, enrichment by collagen I, ALP activity and metabolic shift characteristic for osteogenesis represented a mutually interrelated set of conditions and factors enhancing one another to promote commitment and subsequent terminal differentiation of MSC [26,90,91,93].

According to our results, we can conclude that self-organization of MSC through condensation results in self-patterning influencing their fate. These changes are associated with spatial heterogeneity local microenvironments influencing internal properties of cells. Eventually, populations of cells within condensed areas were primed to osteogenic differentiation prior (e.g., via ALP upregulation and collagen I enrichment) which supported their ability to differentiate to osteocytes under appropriate stimuli.

Seeking for possible mechanisms involved in MSC self-organization, as well as its impact signature at gene expression level we performed RNA-sequencing (RNA-seq) of mRNA isolated from microdissected condensed and scattered areas. Among identified DEGs several groups were highlighted due to their functional roles directly related to tissue repair after injury (Figure 7).

The DEGs with increased expression included a significant number (>50) related to cell proliferation (DNA synthesis, condensation and sister chromatid separation, cytokinesis, etc.) indicating potential increase of proliferation in CS condensed areas. However, this assumption lacks consistency with our data about enhanced MSC differentiation which is typically associated with reduced cell cycling.

Among DEGs with increased expression in condensed areas we annotated a set of genes associated with activity of small Rho GTPases (Figure 8A). Rho GTPases regulate cytoskeletal dynamics, adhesion and coordinate a wide range of processes including migration, cell polarization and cell cycle progression [94,95]. This finding along with cytoskeleton changes observed after phalloidin staining (Figure 2) hinted that activity of Rho GTPases regulated and maintained MSC condensation during their self-organization in CS. Using Y-27632 we confirmed importance of Rho GTPases major downstream effectors—Rho-dependent protein Kinase 1 and 2 (ROCK-1/2) for self-organization of condensed areas within CS (Figure 9). As mentioned before, Rho GTPase pathway via ROCK-1/2 activity governs rearrangement of actin cytoskeleton during mesenchymal condensation preceding organogenesis in vivo [96]. This suggests that cultured MSC retain regulatory pattern and mechanism of condensation similar to their in vivo counterparts [72].

Among upregulated DEGs we did not observe groups of genes that were directly related to osteogenic differentiation enhanced in CS. However, among decreased DEGs, a vast array was controlled by a transcription factor known as sterol regulatory element-binding protein (SREBP) (Figure 8B) creating a fingerprint of its downregulated activity. Targets of SREBP are considered necessary for synthesis and storage of lipids especially in hepatocytes and in cells undergoing adipogenic differentiation. Among them is Fatty acid synthase (FASN) which encodes a multi-enzyme catalyzing the synthesis of the main storage palmitate fatty acid [62,63,97]. Basing on this we suspected involvement of SREBP downregulation induced by MSC condensation.

Activity of SREBP is known to be suppressed by Rho GTPase [64]. Using lipid deposition as a SREBP-dependent endpoint parameter we investigated hypothetic causal link between increased Rho GTPase activity in condensed areas and reduced SREBP-dependent transcription revealed by RNA-seq analysis. We used adipogenic differentiation of MSC in monolayer culture or CS in presence of chemical inhibitors of ROCK-1/2(Y-27632) and SREBP (betulin) (Figure 10). In this experiment changes of lipid accumulation in MSC monolayer culture were consistent with our assumption of Rho-mediated suppression of SREBP. Treatment by Y-27632 increased amount of lipids in monolayer culture while co-inhibition by betulin abolished this effect (Figure 10A). In total CS this response failed to reproduce and we assume this was explained by CS complex composition. The construct contains both condensed and scattered MSC with contrasting status of Rho GTPase pathway and its effects on adipogenic differentiation efficacy. Thus, measurement of lipid accumulation in lysate obtained from whole construct results in “dilution” of effects. Therefore, we proceeded to assessment of local lipid accumulation in condensed areas of CS undergoing adipogenic differentiation. Using fluorescence microscopy and Nile Red lipophilic dye, we demonstrated that inhibition of ROCK-1/2 increased lipid deposition in CS condensed areas while betulin abolished this effect (Figure 10C) which was concordant with our initial understanding of adipogenesis regulation by their targets. Thus, in CS cells reproduce molecular mechanisms responsible for regulation of MSC fate via change of cell state from scattered to condensed and vice versa [98].

To quantify this result, we obtained a reporter cell line to visualize direct effects of inhibitors on expression of adiponectin gene (AdipoQ) characteristic for adipogenic differentiation. Our results showed that during adipogenic differentiation without inhibitors expression of GFP driven by AdipoQ promoter was increased in condensed CS areas compared to both monolayer culture and scattered areas (Figure 11A). Inhibition of ROCK-1/2 resulted in unexpected dramatic drop of GFP signal reflecting reduction of AdipoQ expression in monolayer MSC and, most importantly, in condensed, but not in scattered areas of CS (Figure 11B). Thus, activity of ROCK-1/2 was associated with enhanced adiponectin expression. This was unexpected, since we assumed that regulation of AdipoQ expression was similar to other genes associated with adipogenic differentiation including those driven by SREBP.

Such unexpected effect, however, can be explained knowing that during adipogenesis promoter of AdipoQ (in contrast to FASN), is controlled not only by SREBP, but also by other transcription factors including well-established adipogenesis controller—peroxisome proliferator-activated receptor gamma (PPAR-γ). Medium for adipogenic differentiation contains activators of PPAR-γ such as IBMX which is critical for successful differentiation, so we may suggest a SREBP-independent shunting of pro-adipogenic signal in our model system. For example, well-known molecular pathways may provide a possible explanation of cross-talk between Rho-GTPase, SREBP, PPAR-γ and adiponectin via modulation of AMP-activated protein kinase (AMPK). PPAR-γ is known to be potentiated by PGC-1α which modulates the spectrum of genes activated by this transcription factor. PGC-1α is also a key factor managing metabolic reprogramming of cells after activation of AMPK during adipogenic differentiation. It has been shown that AMPK activity promotes differentiation of preadipocytes into brown fat cells in a PGC-1α-dependent manner [99,100,101,102,103,104,105,106,107,108,109].

Since it is known that Rho-dependent inhibition of SREBP is also mediated by AMPK [64], one may suppose that activation of AMPK by Rho-ROCK-1/2 signaling axis in condensed areas may be responsible for both suppression of SREBP and activation of AdipoQ expression Furthermore, many others of our results are also indicators of increased activity of AMPK in CS condensed areas. For example, AMPK-activated PGC-1a is known (together with NRF-1 and -2) to promote mitogenesis and activation of oxidative phosphorylation [100,102,110,111], AMPK and PGC-1a are also able to enhance enzymatic activity of LDHB (Figure 6) [112]. In addition, AMPK itself is involved in activation of osteogenic transcription factor RUNX which enhances osteogenic potential of MSC via increased ALP content and activity [113,114,115,116,117,118,119,120]. This may explain spontaneous activation of ALP in condensed areas prior to osteogenic differentiation (Figure 4). Increased local deposition of collagen I (Figure 5, lower part) is also concordant with idea of Rho-ROCK-1/2 dependent AMPK activation, since AMPK induces collagen production by many cells, including osteoblasts and MSC [121]. Interestingly, adiponectin also was shown to stimulate osteogenic differentiation of MSC and bone formation [122,123,124,125].

Overall, we may conclude that the activated Rho-ROCK-1/2 axis in condensed areas mediates the observed shifts in the differentiation of MSC and/or their commitment. The phenomenon of autonomous and spontaneous self-organization of MSC by condensation is present in CS. Our data supports its mechanism via the Rho-dependent rearrangement of actin cytoskeleton, which invokes a cascade of signaling and transcription events that result in a higher osteogenic potential and reduction of MSC ability to undergo adipogenesis.

During organogenesis, mesenchymal condensation is involved in the determination of cell fate; therefore, our initial hypothesis that MSC retained an intrinsic ability to determine their fate ex vivo through self-organization has been confirmed. MSC in CS are able to form a spatially heterogeneous system where cells acquire different fates in a compartment-dependent manner, which complies with the definition of self-patterning. This ability of MSCs can manifest as their function during the regeneration of various tissues, e.g., bone—one of the best organs—has a very stringent dependency on MSC condensation, and our study direction may further be expanded to tissue- or niche-specific MSC to seek potential ways for more effective or faster regeneration in human.

## Figures and Tables

**Table 1:** Brief overview of the microscopy procedures and equipment used in the respective methods and assays.

Method or Assay	Procedure	Equipment	Notes
Cell sheet assembly procedure; Adiponectin reporter assay and ROCK-1/2 inhibitory analysis	Long-term microscopic observations of the cell sheet assembly and fluorescence assays (coculture with endothelium or differentiation experiments)	Nikon ECLIPSE Ti microscope (Nikon, Tokyo, Japan)	Monitoring of live cells in culture gas mixture, temperature and humidity conditions suitable for long-term growth. Photocapture was carried out for 12–16 days, with a frequency of shooting each 40 min using the control software Nikon program NIS—Elements Advanced (Nikon, Tokyo, Japan)
Images or videos processing in all cases	Image and videos processing in all experiments	Fiji plugin collection for NIH ImageJ (1.53c version, NIH, Bethesda, MD, USA) [33]	
Cell culture hematoxylin staining in cultures dishes and plates; Adipogenic, osteogenic, and chondrogenic differentiation of MSC; Alkaline phosphatase activity assay	Acquisition and counts of condensed areas; assessment of the differentiation at the endpoint; visualization of ALP activity after incubation with chromogenic substrates	Nikon SMZ18 (Nikon, Tokyo, Japan) stereomicroscope	Surface and number of condensed areas in each well were measured using unified thresholding and binarization procedures in Fiji plugin collection for NIH ImageJ (1.53c version, NIH, Bethesda, MD, USA)
Immunofluorescent labeling of α-smooth muscle actin (α-SMA) and Phalloidin fluorescent staining; Adipogenic, osteogenic and chondrogenic differentiation of MSC; Alkaline phosphatase activity assay; MitoTracker™ probing for mitochondrial transmembrane potential; Adiponectin reporter assay and ROCK-1/2 inhibitory analysis	Visualization and image acquisition	Leica DMi8 inverted fluorescence microscope with a DFC7000T camera (Leica Microsystems, Wetzlar, Germany).	Additional image processing and analysis was carried out in the Fiji plugin collection for NIH ImageJ (1.53c version, NIH, Bethesda, MD, USA)

## Data Availability

The data that support the findings of this study are available from the corresponding author upon reasonable request.

## Supplementary Materials

All supplementary materials are available online at https://doi.org/10.5281/zenodo.5498128.
